# Supplementary material for: Reconciling Mining with the Conservation of Cave Biodiversity: A Quantitative Baseline to Help Establish Conservation Priorities
Source: PLoS One. 2016 Dec 20;11(12):e0168348. doi: 10.1371/journal.pone.0168348 (PMC5173368; doi:10.1371/journal.pone.0168348)
Supplement: S1 Dataset — (ZIP) [file pone.0168348.s002.zip › Taxa/Serra Norte/SN_2013/N5S/Lista N5S-75.pdf]

CAVIDADE N5S-0075

Classe	Ordem	Fam/Outros	Gên/Outros	Espécie	S-U	U-S	Zon.
Arachnida	Acari	(Oribatida)		sp.6		X	E
Arachnida	Acari	(Parasitengonina)		sp.7		X	P
Arachnida	Acari	Ascidae		sp.1		X	P
Arachnida	Acari	Eupodidae		sp.1	X		P
Arachnida	Acari	Ixodidae		jovem	X		P
Arachnida	Acari	Laelapidae		sp.73		X	P
Arachnida	Acari	Podocinidae	*Podocinum*	sp.1		X	E
Arachnida	Acari	Rhagidiidae		sp.5		X	P
Arachnida	Acari	Rhagidiidae		sp.20	X		P
Arachnida	Acari	Trombiculidae		sp.39	X		P
Arachnida	Amblypygi	Charinidae	*Charinus*	sp.1		1	P
Arachnida	Amblypygi	Phrynidae	*Heterophrynus*	*longicornis*	4	3	P
Arachnida	Araneae	Araneidae		jovem	X	X	E/P
Arachnida	Araneae	Caponiidae		sp.1	X	X	P
Arachnida	Araneae	Corinnidae		jovem		1	P
Arachnida	Araneae	Ctenidae		jovem	1		E
Arachnida	Araneae	Drymusidae	*Drymusa*	*spelunca*	X	X	P
Arachnida	Araneae	Ochyroceratidae	*Speocera*	sp.1	X	X	P
Arachnida	Araneae	Pholcidae	*Leptopholcus*	sp.1		X	P
Arachnida	Araneae	Pholcidae	*Mesabolivar*	sp.1	X		P
Arachnida	Araneae	Pisauridae		jovem	1		E
Arachnida	Araneae	Scytodidae	*Scytodes*	sp.1	5		P
Arachnida	Araneae	Tetrablemmidae		jovem		X	E
Arachnida	Araneae	Theraphosidae	aff. *Holothele*	sp.1	1	2	E
Arachnida	Araneae	Theridiidae	*Theridion*	sp.1	X	X	P
Arachnida	Araneae	Theridiosomatidae	*Plato*	sp.1	X	X	P
Arachnida	Opiliones	Escadabiidae		sp.1		X	P
Arachnida	Opiliones	Stygnidae	*Protimesius*	*gracilis*		1	P
Arachnida	Palpigradi	Eukoeneniidae	*Allokoenenia*	sp.1		X	E/P
Arachnida	Palpigradi	Eukoeneniidae	*Eukoenenia*	sp.1		X	E/P
Arachnida	Pseudoscorpiones	Bochicidae		jovem	X	X	E/P
Arachnida	Pseudoscorpiones	Chernetidae		sp.1	X		P
Arachnida	Pseudoscorpiones	Chthoniidae	*Pseudochthonius*	sp.1	X	X	E/P
Arachnida	Ricinulei	Ricinoididae	*Cryptocellus*	jovem		1	E
Chilopoda	Geophilomorpha	Ballophilidae	*Ityphilus*	sp.1	1		P
Chilopoda	Scolopendromorpha	Cryptopidae	*Cryptops*	jovem		1	P
Chilopoda	Scolopendromorpha	Scolopocryptopidae	*Newportia*	sp.1		1	P
Diplopoda	Polydesmida	Chelodesmidae		jovem		3	P
Diplopoda	Polydesmida	Fuhrmannodesmidae		jovem		X	E/P
Diplopoda	Polydesmida	Pyrgodesmidae		sp.1	X	X	P
Entognatha	Collembola	Isotomidae		sp.1		X	P
Entognatha	Collembola	Paronellidae		sp.1	X	X	E/P
Entognatha	Collembola	Poduromorpha		sp.1		X	E
Entognatha	Collembola	Sminthuroidea		sp.2		X	E/P
Entognatha	Diplura	Campodeidae		sp.1		X	E
Entognatha	Diplura	Japygidae		sp.1	X		P
Insecta	Blattodea			jovem		1	E
Insecta	Coleoptera	Carabidae		sp.3	X	X	P
Insecta	Coleoptera	Ptiliidae		jovem	X		P

Insecta	Coleoptera	Scydmaenidae		sp.2	X		P
Insecta	Coleoptera	Staphylinidae	Pselaphinae	sp.5		X	P
Insecta	Coleoptera	Staphylinidae		sp.1		X	E
Insecta	Diptera	Cecidomyiidae	Cecidomyiinae	sp.		X	E/P
Insecta	Diptera	Ceratopogonidae		sp.		X	P
Insecta	Diptera	Chaoboridae		sp.		X	E
Insecta	Diptera	Chironomidae		sp.	X		P
Insecta	Diptera	Dolichopodidae		sp.	X		E
Insecta	Diptera	Phoridae		sp.	X		P
Insecta	Diptera	Psychodidae	Phlebotominae	sp.	X	X	P
Insecta	Diptera	Tipulidae	Tipulinae	sp.		X	E
Insecta	Heteroptera	Dipsocoroidea		sp.	X		P
Insecta	Heteroptera	Reduviidae	Emesinae	sp.1	1	9	P
Insecta	Heteroptera	Reduviidae	Triatominae	sp.	1		
Insecta	Heteroptera	Tingidae		sp.	X		P
Insecta	Homoptera	Cixiidae		jovem	X	X	P
Insecta	Hymenoptera	Formicidae	*Gnamptogenys*	sp.1		X	E
Insecta	Hymenoptera	Formicidae	*Hypoponera*	sp.1	X	X	E/P
Insecta	Hymenoptera	Formicidae	*Pachycondyla*	sp.2	X	X	E/P
Insecta	Hymenoptera	Formicidae	*Paratrechina*	sp.1		X	P
Insecta	Hymenoptera	Formicidae	*Trachymyrmex*	sp.1		X	E
Insecta	Isoptera	Termitidae	*Nasutitermes*	sp.	X		E
Insecta	Isoptera	Termitidae	*Planicapritermes*	sp.	X	X	P
Insecta	Isoptera	Termitidae	*Termes*	sp.	X		P
Insecta	Lepidoptera	Noctuoidea		sp.5		1	E
Insecta	Lepidoptera	Tineoidea		sp.1	X		P
Insecta	Orthoptera	Mogoplistidae	*Cycloptiloides*	*americanus*	1	1	E
Insecta	Orthoptera	Phalangopsidae	*Paraclodes*	jovem	30	2	E/P
Insecta	Orthoptera	Phalangopsidae	*Phalangopsis*	jovem	3	25	P
Insecta	Psocoptera	Ptiloneuridae	*Triplocania*	sp.4	X	X	P
Gastropoda	Pulmonata	Subulinidae		sp.2		X	P
Gastropoda	Pulmonata	Systrophiidae	*Happia*	sp.		X	E
Malacostraca	Isopoda	Philosciidae		sp.2		X	E
Malacostraca	Isopoda	Scleropactidae	*Circoniscus*	*buckupi*	X		P
Symphyla		Scutigerellidae		sp.	X	X	P
Amphibia	Anura	Strabomantidae	*Pristimantis*	cf. *fenestratus*	2		E
